# A simple, no-cost method of preventing contamination of anaesthesia work area

**DOI:** 10.4103/0019-5049.72664

**Published:** 2010

**Authors:** Ravi L Bhat, Harihar V Hegde, P Raghavendra Rao

**Affiliations:** Department of Anaesthesiology, SDM College of Medical Sciences and Hospital, Sattur, Dharwad, India

Sir,

It is well known that contamination of the anaesthesia work area with potential bacterial pathogens and blood occurs intraoperatively[1] following general anaesthesia. It has been demonstrated that bacterial contamination occurs early (within as little as 4 min) and is unrelated to factors of case duration, urgency, or patient American Society of Anaesthesiologists physical status. Contamination with saliva represents a potential risk, since saliva is the main vehicle of infection for nonparenteral transmission of hepatitis-B.[2] Bacterial transfer to patients is associated with the variable aseptic practice of anaesthesia personnel. Placing the laryngoscope blade in a container (e.g. a kidney tray) following intubation with subsequent contamination of the anaesthesia work area is unwanted but not an uncommon feature in the operation theatre.

**Figure 1:**
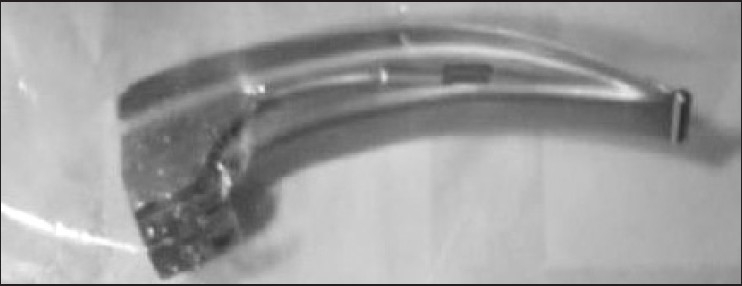
Quarantined laryngoscope blade. Used laryngoscope blade inside the plastic cover of the disposable PVC endotracheal tube

We propose the use of the plastic cover of the disposable PVC endotracheal tube to keep the contaminated laryngoscope blade to avoid soiling the top of the anaesthesia machine and work area. The laryngoscope blade can easily be kept inside this plastic cover which had been opened before intubation [Figure 1]. The contaminated blade can then safely be transported and cleansed without leading to any untoward contamination, and the plastic cover safely discarded. One needs to be careful that the two sides of the cover (one plastic, the other paper) do not come apart during the whole procedure, lest it contaminate other unwanted areas.

The complex intraoperative environment has been theoretically associated with the development of nosocomial infections and may contribute to the emerging pattern of increasing bacterial resistance in the hospital setting. Currently, there is no consensus regarding a satisfactory method for the routine cleaning, disinfection and sterilization of laryngoscope blades and handles. It has been shown that 33% of anaesthesia work surfaces[3] and 38% of laryngoscope blades are contaminated with blood with a high proportion of both blades and handles showing evidence of microbial contamination.[4] Tobin MJ and colleagues[5] suggested the use of commercially available plastic to cover the laryngoscope handle during use to prevent contamination. This involves extra effort and cost; also, laryngoscope blade is the most contaminated are rather than the handle. We think that it is more appropriate to handle the contaminated blade carefully to prevent cross-contamination

Our method of safeguarding the anaesthesia work area from soiling with the contaminated laryngoscope blade is very simple, does not involve any extra expenditure and has the potential to reduce iatrogenic transmission of infection in the perioperative setting.
